# Malaria Test, Treat and Track policy implementation in Angola: a retrospective study to assess the progress achieved after 4 years of programme implementation

**DOI:** 10.1186/s12936-020-03338-x

**Published:** 2020-07-20

**Authors:** Sergio C. Lopes, Rukaaka Mugizi, João Esteves Pires, Fernando David, José Martins, Pedro Rafael Dimbu, Filomeno Fortes, Joana Rosário, Richard Allan

**Affiliations:** 1 PMI Eye Kutoloka Project, The MENTOR Initiative, Haywards Heath, UK; 2 National School of Public Health, Luanda, Angola; 3 PMI Eye Kutoloka Project, World Learning, Luanda, Angola; 4 National Malaria Control Programme, Luanda, Angola

**Keywords:** Malaria, Supervision, Angola, Case management, Test, Treat, Track

## Abstract

**Background:**

Malaria is one of the main causes of death in Angola, particularly among children under 5 years of age. An essential means to improve the situation is with strong malaria case management; this includes diagnosing suspected patients with a confirmatory test, either with a rapid diagnostic test (RDT) or microscopy, prompt and correct treatment with artemisinin-based combination therapy (ACT), and proper case registration (track). In 2011, the United States President’s Malaria Initiative (PMI) launched a country-wide programme to improve malaria case management through the provision of regular training and supervision at different levels of health care provision. An evaluation of malaria testing, treatment and registration practices in eight provinces, and at health facilities of various capacities, across Angola was conducted to assess progress of the national programme implementation.

**Methods:**

A retrospective assessment analysed data collected during supervision visits to health facilities conducted between 2012 and 2016 in 8 provinces in Angola. The supervision tool used data collected for malaria knowledge, testing, treatment and case registration practices among health workers as well as health facilities stock outs from different levels of health care delivery. Contingency tables with Pearson chi-squared (*χ*^2^) tests were used to identify factors associated with “knowledge”, “test”, “treat” and “track.” Multivariable logistic regression models were used to assess factors associated with the defined outcomes.

**Results:**

A total of 7156 supervisions were conducted between September 2012 and July 2016. The overall knowledge, testing, treatment and tracking practices among health care workers (HCWs) increased significantly from 2013 to 2016. Health care workers in 2016 were 3.3 times (95% CI: 2.7–3.9) as likely to have a higher knowledge about malaria case management as in 2013 (*p* < 0.01), 7.4 (95% CI: 6.1–9.0) times as likely to test more suspected cases (*p* < 0.01), 10.9 (95% CI: 8.6–13.6) times as likely to treat more confirmed cases (*p* < 0.01) and 3.7 (95% CI: 3.2–4.4) times as likely to report more accurately in the same period (*p* < 0.01).

**Discussion:**

Improvements demonstrated in knowledge about malaria case management, testing with RDT and treatment with artemisinin-based combinations among HCWs is likely associated with malaria case management trainings and supportive supervisions. Gaps in testing and treatment practices are associated with RDT and ACT medicines stock outs in health facilities. Tracking of malaria cases still poses a major challenge, despite training and supervision. Hospitals consistently performed better compared to other health facilities against all parameters assessed; likely due to a better profile of HCWs.

**Conclusion:**

Significant progress in malaria case management in eight provinces Angola was achieved in the period of 2013–2016. Continued training and supportive supervision is essential to sustain gains and close existing gaps in malaria case management and reporting in Angola.

## Background

Despite significant progress in the past 2 decades, Africa still accounts for 90% of malaria deaths worldwide with higher incidence in children less than five years of age [1]. Together with vector control, prompt and correct case management and correct reporting play a significant role in reducing malaria morbidity and mortality.

In 2012, the World Health Organization (WHO) launched *T3: Test. Treat. Track.* initiative to ensure all suspected malaria cases were properly tested, treated and registered [2]. To evaluate this policy, it is important to analyse each of its elements separately and understand the main drivers and bottlenecks to achieve good results in T3 policy. Reliable stock management of RDTs and laboratory supplies allied to adequate training and supervision was found to be of great importance to ensure high testing rates of suspected malaria cases [3–7]. Non-adherence to malaria RDT or microscopy results, particularly when results are negative and presumptive treatment of febrile cases are treated as malaria cases is associated with over prescription of unnecessary malaria drugs [8–14]. Incorrect diagnosis and treatment practices were found to be related with health workers´ knowledge [15, 16] and their perceptions and beliefs regarding malaria diagnosis [17, 18] and RDT results [10, 19, 20], patients’ clinical and demographic characteristics [21, 22], and also with patients’ expectations and demands [9, 18, 23]. Similarly, continuous and reliable provision of ACT medicines seems to be associated with improved malaria treatment practices [24, 25]. The role of training and supervision was also identified to be an important factor to improve malaria testing and treatment practices [5, 13, 15, 26]. However, in some studies this human resource support was not found to be associated with improved malaria case management [27, 28].

In Angola, malaria represents one of the major public health problems accounting for a 5th of all inpatients in public health facilities [29]. Ensuring quick and adequate diagnosis and treatment of all malaria cases is one of the strategies adopted by Angolan National Malaria Control Programme (NMCP) to reduce malaria burden. The use of ACT, free of charge in the public sector, was adopted in 2004 [29]. Training and supervision initiatives targeting the introduction of malaria RDT and ACT started in 2006 [13]. Mandatory testing before treatment policy was set in 2009 [25].

In 2011, US President’s Malaria Initiative (PMI) funded a programme to improve malaria case management in eight provinces in Angola: Uíge, Zaire, Kwanza Norte, Kwanza Sul, Malanje, Benguela, Huila and Huambo. The programme focused on providing extensive training to health workers coupled with regular supportive and formative supervision visits to municipal departments of health and health facilities. Training of laboratory technicians and warehouse managers was also conducted to improve quality of diagnosis and provision of RDT and ACT [30].

This paper aims to describe the trends of uncomplicated malaria case management in targeted provinces throughout the programme implementation by analysing data collected during supportive supervision visits conducted in health facilities. Uncomplicated malaria case management is assessed by composite indicators of (1) suspected cases tested; (2) positive cases treated with first-line recommended anti-malarial; and (3) correctness of malaria reporting by health facilities (Test. Treat. Track). Two other components were also assessed: malaria knowledge amongst health care workers supervised and RDT/ACT medicine stock-outs.

## Methods

### Study site and design

Retrospective analysis of data from 7156 supportive supervisions was performed. Supervision, supported by PMI, was conducted between September 2012 and July 2016 in 8 provinces across Northern and Central Angola: Benguela, Huambo, Huíla, Kwanza Norte, Kwanza Sul, Malanje, Uige, Zaire. Three levels of health systems were continuously monitored throughout the study period: hospitals (tertiary care), health centres (secondary care) and health posts (primary care).

### Supervision

#### Data collection

Joint supportive supervision was conducted by local malaria focal person from the Ministry of Health (MoH) together with a pool of trained supervisors from different non-governmental organizations (NGOs) working under the PMI framework across the different provinces. Supervisors were trained for 2 days on the use of the supervision guide tool. Training was performed by Central level NMCP staff.

NGO supervisors were responsible for data collection and were trained and routinely supervised for this task to ensure adequate data collection. HF supervision plans were established on a monthly basis with local health authorities, according to the needs and staff availability. The programme aimed to visit each HF, at least once a year. HCW or HFs identified to have low performance were visited more regularly. In Huambo, the program was discontinued by the end of Fiscal Year 2015 and no data was collected in this province in FY 2016. World Learning, an NGO, provided technical assistance and coordinated all PMI supported NGOs.

### Supervision guide

For the purpose of data collection, a standardized supervision guide was used by the supervisors at each visit. The guide was elaborated based on NMCP Supervision Guide to Health Units [31] and adopted by the PMI supported NGOs. It comprised eight indicators aiming to assess: health care worker malaria knowledge; malaria testing (“Test”) and treating practices (“Treat”); quality of malaria reporting (“Track”) and health facility stock-outs. Details on each indicator and the criteria used to classify each one of them are explained in the Additional file 1. Knowledge questions were performed on each HCW in each selected HF. The supervised HCW had to be working in outpatient clinic and managing malaria cases. Therefore, knowledge indicators presented refer to a single HCW in each facility even when HF had more than one staff performing malaria case management activities. Testing, treating and tracking indicators were found through health facility patient register checks and refer to health facility performance (more than one HCW in most of cases).

Questions 1–4 aimed to assess HCW knowledge on malaria prevention and case management: Q1 (HCW knows how to perform RDT); Q2 (knowledge on fever differential diagnosis); Q3 (knowledge on ACT dosage and posology); Q4 (knowledge on IPTp-SP starting date). Questions were made directly to HCW and correction and clarifications given when answers were incorrect. After questioning, demonstrations were requested to the HCW or performed by the supervisor to explain correct procedures for RDT, fever differential diagnosis and ACT posology. Question 5 was designed to assess testing rates by measuring the proportion of tested malaria suspected cases (“Test”). Question 6 aimed to evaluate adequacy of treatment of malaria confirmed cases by assessing the proportion of confirmed cases receiving ACT (“Treat”). Question 8 assessed correctness of HF monthly malaria reports against health facility patient registers (“Track”). One question focused on stocks of ACT medicines and RDT (Question 7). Stock outs were considered if the HF registered a period of 7 or more days without ACT/RDT in the past 3 months. Indicators 5, 6 and 8 were assessed by retrieving and analysing HF patient register data. Review of pharmacy stocks was performed during supervision and data was used to respond to Question 7.

For questions 1–4, a binomial variable (knows/doesn’t know) was developed. For questions 5 and 6, categories defined in the questionnaire were computed to create a binomial variable with a cut-off point of 75% of suspected cases tested (Question 5) and 75% of non-severe cases treated with ACT (Question 6). The 75% cut off point was used as data collection was based on pre-defined NMCP supervision guidance tools which did not collect data using continuous values. The 75% + interval was used as it was the higher interval of the pre-set NMCP categories. For question 7, three possible values were considered at the moment of supervision: No stock-outs, stock-out of ACT medicines, or stock-out of RDT. For question 8, only 2 values were assumed as possible: Agreement vs Disagreement of health facility patient register with the HF monthly malaria report for the same period. Number of cases and number of deaths were counted from HF register books and totals compared with monthly report. In case of any discrepancy, the team would assume a 'disagreement'. The tool was subject to pilot and review during the first quarter of implementation. Criteria for HCW assessment in each question was included to ensure standardization between different supervisors and different locations.

It is important to emphasize that Years correspond in this study to Fiscal Year between October and September. Therefore, when reading 2013, it should be considered it corresponds to the year between October 2012 and September 2013. The same applies for 2014, 2015 and 2016. For 2016, there is no data on Huambo province as the program was discontinued.

### Data management and statistics

Data was double entered into a Microsoft Excel database by monitoring and evaluation officers in the eight provinces. Data was then compiled and checked for consistency and correctness at Central level by World Learning monitoring and evaluation officer. Analysis was conducted using STATA/SE v.12 software.

A composite variable denominated “knowledge” was defined by having answered correctly to questions 1–4. A binary result was used to report this variable: “Yes” for those correctly answering the four questions and “No” for those failing to correctly answer one or more questions. Contingency tables with Pearson chi-squared (*χ*^2^) tests were used to identify factors associated with the “knowledge” composite variable, “test”, “treat” and “track”. Pertinent variables (Year, Province and Health Facility Type, RDT and ACT stock-outs) with a significant *χ*^2^ statistic for the association with outcome variables were used to build four multivariable logistic regression models for “knowledge”, “test”, “treat” and “track”, respectively. Estimated prevalence and adjusted odds ratios (OR) are reported with corresponding 95% confidence intervals (95% CI).

### Ethical considerations

All supervisions were previously authorized by NMCP and the respective provincial and municipal health departments. Data was analysed and shared with provincial and municipal level authorities to support planning and decision making. No personal information from HCWs was collected.

## Results

### Distribution of supervisions by time and location

A total of 7156 supervisions were conducted between September 2012 and July 2016. Health posts accounted for 73.4% of total supervisions, followed by health centres (23.5%) and hospitals (3.1%). More supervisions were conducted in 2015 (2178) than in 2013 (1335), 2014 (1882) and 2016 (1761) (Table 1). Uige province registered the largest proportion of supervisions conducted in the 4 years (18.4%), followed by Benguela (17.2%), Kwanza Norte (12.6%), Zaire (11.8%), Huambo (11.1%), and Huila (10.6). Malanje and Kwanza Sul accounted for less than 10% of the total supervisions conducted. The distribution of supervisions per province per year was balanced, apart from 2013 when a smaller proportion of supervisions were conducted in all provinces except Huambo (Table 2).

**Table 1:** Distribution of number of supervisions per province, per year

	Year, n (%)				
	2013	2014	2015	2016	Total
Province					
Benguela	239 (19.4%)	272 (22.1%)	376 (30.5%)	346 (28.1%)	1233
Huambo	294 (37.1%)	222 (28.0%)	276 (34.8%)	0 (0%)	792
Huila	48 (6.3%)	157 (20.6%)	201 (26.4%)	355 (46.6%)	761
Kwanza Norte	227 (25.2%)	230 (25.5%)	224 (24.9%)	220 (24.4%)	901
Kwanza Sul	51 (7.8%)	200 (30.6%)	220 (33.6%)	183 (28.0%)	654
Malange	68 (10.3%)	226 (34.3%)	226 (34.3%)	138 (21.0%)	658
Uige	231 (17.6%)	371 (28.2%)	384 (29.2%)	330 (25.1%)	1316
Zaire	177 (21.0%)	204 (24.3%)	271 (32.2%)	189 (22.5%)	841
Health facility					
Health posts	975 (18.6%)	1358 (25.9%)	1607 (30.6%)	1311 (24.9%)	5251
Health centres	313 (18.6%)	457 (27.2%)	507 (30.1%)	406 (24.1%)	1683
Hospitals	47 (21.2%)	67 (30.2%)	64 (28.8%)	44 (19.8%)	222
Total	1335 (18.7%)	1882 (26.3%)	2178 (30.4%)	1761 (24.6%)	7156

**Table 2:** Results of a multivariable regression model with knowledge about malaria case management as outcome

Knowledge	Associated factor	% Correct (95% CI)	Adjusted OR (95% CI)
	Year		
	2013	49.3 (46.6–52.0)	–
	2014	53.2 (51.0–55.5)	1.7 (1.4–2.0)
	2015	59.5 (57.4–61.5)	2.2 (1.8–2.5)
	2016	62.0 (59.7–64.3)	3.3 (2.7–3.9)
	Province		
	Benguela	74.5 (71.9–76.9)	9.7 (7.7–12.2)
	Huambo*	78.0 (75.0–80.9)	15.6 (12.1–20.2)
	Huila	45.9 (42.3–49.5)	2.3 (1.8–2.9)
	Kwanza Norte	50.4 (47.1–53.7)	3.8 (3.0–4.7)
	Kwanza Sul	24.3 (21.1–27.8)	–
	Malanje	70.8 (67.2–74.3)	8.1(6.3–10.4)
	Uige	29.9 (27.4–32.4)	1.4 (1.1–1.7)
	Zaire	82.1 (79.3–84.6)	16.0 (12.4–20.7)
	Health facility type		
	Health posts	52.9 (51.6–54.3)	–
	Health centres	66.0 (63.7–68.3)	1.4 (1.2–1.6)
	Hospitals	70.3 (63.8–76.2)	1.6 (1.2–2.2)

All *p* values < 0.05

^*^From 2013 to 2015 only in Huambo

### Knowledge

**Fig. 1:**
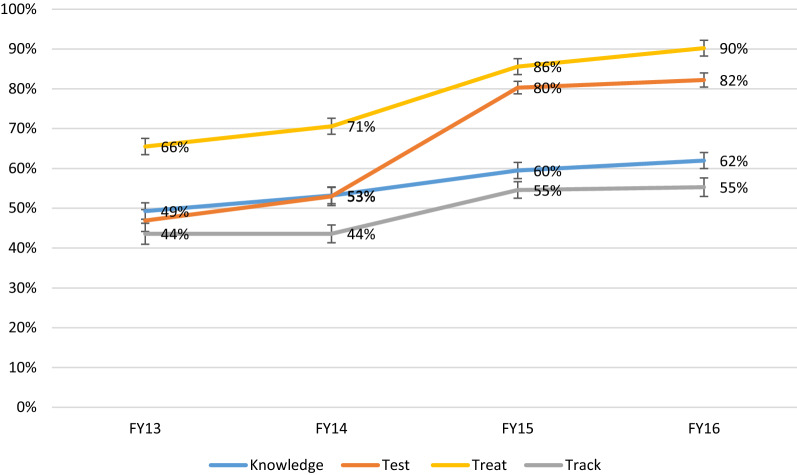
Evolution of malaria Knowledge, Test, Treat and Track in 8 provinces in Angola. For 2016, there is no data on Huambo

The overall knowledge among HCW increased significantly throughout the time of the analysis from 49.3% (95% CI: 46.6–52.0) in 2013 to 62.0% (95% CI: 59.7–64.3) in 2016 (Fig. 1). Multivariate analysis showed that HCWs in 2016 had 3.3 (95% CI: 2.7–3.9) times the odds to have a higher composite score of malaria case management knowledge than workers in 2013 (*p* < 0.01) (Table 2).

There was a significant difference when looking at the knowledge level across the different provinces, with Kwanza Sul [24.3% (95% CI: 21.1–27.8)] and Uíge 29.9% (95% CI: 27.4–32.4) showing the lowest proportions, whereas Zaire [82.1% (95% CI: 79.3–84.6) and Huambo 78.0% (95% CI: 75.0–80.9)] showed the highest. Multivariate analysis showed that HWs in Zaire had 16.0 (95% CI: 12.4–20.7) times the odds of having higher knowledge than workers in Kwanza Sul (*p* < 0.01).

Healthcare workers based in the Hospitals had a higher overall level of knowledge when compared with the ones working in the health posts [70.3% (95% CI: 63.8–76.2) vs 52.9% (95% CI: 51.6–54.3)]. In fact, Hospital based HCWs had 1.6 (95% CI: 1.2–2.2) times the odds of having a higher knowledge than workers in health posts after controlling for confounding (*p* < 0.01).

### Test

The proportion of health facilities testing by RDT 75% or more of malaria suspected cases increased significantly over the time of the analysis from 46.9% (95% CI: 44.2–49.6) in 2013 to 82.2% (95% CI: 80.3–83.9) in 2016 (Table 3). Multivariate analysis showed that HCW in 2016 had 7.4 (95% CI: 6.1–9.0) times the odds of testing more suspected cases than in 2013 (*p* < 0.01).

**Table 3:** Results of a multivariable regression model with testing 75% or more malaria suspected cases as outcome

Test	Associated factor	% Correct (95% CI)	Adjusted OR (95% CI)
	Year		
	2013	46.9 (44.2–49.6)	–
	2014	53.0 (50.7–55.3)	1.4 (1.2–1.7)
	2015	80.3 (78.6–82.0)	5.1 (4.3–6.1)
	2016	82.2 (80.3–83.9)	7.4 (6.1–9.0)
	Province		
	Benguela	79.2 (76.8–81.4)	4.9 (4.0–6.0)
	Huambo*	72.2 (69.0–75.3)	4.7 (3.8–5.8)
	Huila	72.4 (69.1–75.6)	2.2 (1.8–2.8)
	Kwanza Norte	84.0 (81.5–86.4)	10.1 (7.9–12.8)
	Kwanza Sul	53.2 (49.3–57.1)	1.2 (1.0–1.5)
	Malanje	76.0 (72.5–79.2)	3.8 (3.0–4.8)
	Uige	45.7 (43.0–48.4)	–
	Zaire	61.2 (57.8–64.5)	1.9 (1.5–2.3)
	Health facility		
	Health posts	65.9 (64.7–67.3)	–
	Health centres	69.9 (64.7–67.3)	1.4 (1.2–1.6)
	Hospitals	80.2 (74.3–85.2)	1.8 (1.2–2.7)
	Stocks RDT		
	Available	77.6 (76.4–78.7)	3.4 (3.0–3.8)

All *p* values < 0.05

^*^From 2013 to 2015 only in Huambo

There was a significant difference when looking at the testing practices across the different provinces, with Kwanza Sul [53.2% (95% CI: 49.3–57.1)] and Uíge [45.7% (95% CI: 43.0–48.4)] showing the lowest proportions, whereas Kwanza Norte (84.0% (95% CI: 81.5–86.4) showed the highest. Multivariate analysis showed that HFs in Kwanza Norte had 10.1 (95% CI: 7.9–12.8) times to the odds of testing more suspected malaria cases than HCWs in Uige (*p* < 0.01).

The proportion of tested malaria suspected cases was higher in Hospitals than in health posts (80.2% (95% CI: 74.3–85.2) vs 65.9% (95% CI: 64.7–67.358.8). In fact, suspected malaria cases in Hospitals were 1.8 (95% CI: 1.2–2.7) times more likely to be test than in health posts (*p* < 0.01).

**Fig. 2:**
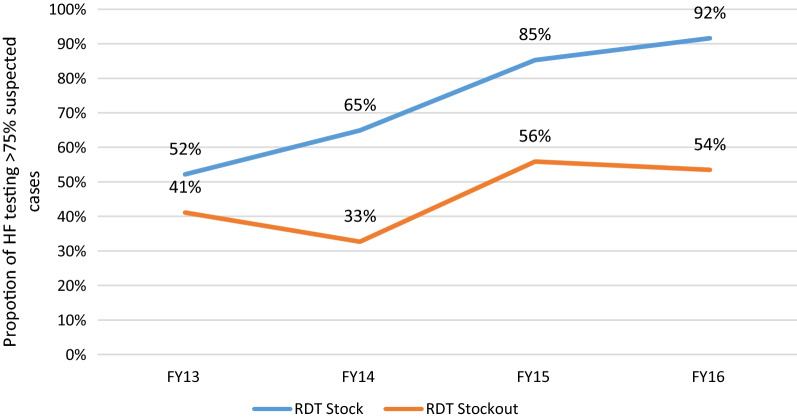
Proportion of health facilities testing > 75% of suspected cases tested per FY (RDT stock out vs no stock out)

The existence of RDT stocks is strongly associated with the HF capacity to test the majority of suspected malaria cases (Adj OR: 3.4; 95% CI: 3.0–3.8, *p* < 0.01). This trend was visible throughout the program implementation period where HFs were consistently testing a higher proportion of malaria suspected cases (Fig. 2).

### Treat

The proportion of health facilities treating 75% or more malaria confirmed cases with recommended artemisinin-based combinations increased significantly throughout the time of the analysis from 65.5% (95% CI: 62.8–68.0) in 2013 to 90.2% (95% CI: 88.8–91.6) in 2016 (Table 4). Multivariate analysis showed that HFs in 2016 had 10.9 (95% CI: 8.6–13.6) times the odds of treating more confirmed cases than in 2013 (*p* < 0.01).

**Table 4:** Results of a multivariable regression model with treating 75% or more of confirmed malaria cases as outcome

Treat	Associated factor	% Correct (95% CI)	Adjusted OR (95% CI)
	Year		
	2013	65.5 (62.8–68.0)	–
	2014	70.6 (68.5–72.7)	1.9 (1.6–2.3)
	2015	85.6 (84.0–87.0)	5.4 (4.4–6.5)
	2016	90.2 (88.8–91.6)	10.9 (8.6–13.6)
	Province		
	Benguela	96.2 (95.0–97.2)	15.4 (10.8–21.0)
	Huambo*	83.1 (80.3–85.6)	5.1 (3.9–6.7)
	Huila	71.2 (67.7–74.3)	–
	Kwanza Norte	93.1 (91.3–94.7)	9.4 (6.7–13.0)
	Kwanza Sul	66.7 (62.9–70.3)	1.2 (0.9–1.5)*
	Malanje	83.1 (80.0–85.9)	2.8 (2.1–3.7)
	Uige	62.6 (59.9–65.2)	1.0 (0.8–1.3)*
	Zaire	74.3 (71.2–77.2)	1.5 (1.2–2.0)
	Health facility		
	Health posts	78.1 (77.0–79.2)	–
	Health centres	81.2 (79.2–83.0)	1.3 (1.1–1.5)
	Hospitals	84.7 (79.3–89.2)	1.4 (0.9–2.1)*
	Stocks ACT		
	Available	83.5 (82.5–84.5)	3.1 (2.7–3.6)

All *p* values < 0.05; *p > 0.05

^*^From 2013 to 2015 only in Huambo

Differences in treatment practices varied across provinces, with Kwanza Sul [66.7% (95% CI: 62.9–70.3)] and Uíge [62.6% (95% CI: 59.9–65.2)] showing the lowest proportions, whereas Benguela [96.2% (95% CI: 95.0–97.2)] and Kwanza Norte [93.1% (95% CI: 91.3–94.7)] showed the highest. Multivariate analysis showed that HFs in Benguela were 15.4 (95% CI: 10.8–21.0) times more likely to treat confirmed malaria cases with appropriate ACT medicines as HCWs in Huila (*p* < 0.01).

The proportion of HFs treating adequately higher proportions of confirmed cases of malaria was higher in Hospitals than in health posts [84.7% (95% CI: 79.3–89.2) vs 78.1% (95% CI: 77.0–79.2)]. However, these differences were not statistically significant when using multivariate analysis.

**Fig. 3:**
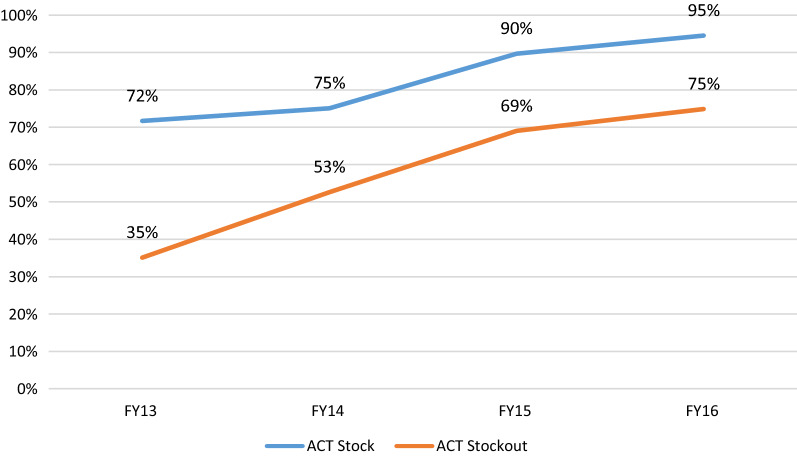
Proportion of health facilities treating > 75% of uncomplicated malaria cases with approved ACT per FY (ACT stock out vs no stock out)

The existence of ACT medicines in stock is associated with HF capacity to treat the majority of confirmed malaria cases with adequate ACT (Adj OR: 3.1; 95% CI: 2.7–3.7, *p *< 0.01). As it is visible in Fig. 3, HFs with ACT medicine stocks consistently treated adequately a higher proportion of malaria confirmed cases (Fig. 3).

### Track

Malaria case reporting accuracy increased throughout the time of the analysis from 43.6% (95% CI: 40.9–46.3) of reports matching the Health Facility patient registers in 2013 to 55.3% (95% CI: 53.0–57.6) in 2016 (Table 5). Despite multivariate analysis results showing that malaria reports in 2016 were 3.7 (95% CI: 3.2–4.4) times more likely to be accurate than in 2013 (*p* < 0.01), it is possible to observe that reporting accuracy improvements throughout time were consistently lower than the other indicators measured (Fig. 1).

**Table 5:** Results of a multivariable regression model with accuracy in malaria case reporting as outcome

Track	Associated factor	% Correct (95% CI)	Adjusted OR (95% CI)
	Year		
	2013	43.6 (40.9–46.3)	–
	2014	43.6 (41.3–45.8)	1.7 (1.5–2.0)
	2015	54.6 (52.5–56.7)	2.8 (2.4–3.3)
	2016	55.3 (53.0–57.6)	3.7 (3.2–4.4)
	Province		
	Benguela	70.2 (67.5–72.7)	8.6 (6.9–10.8)
	Huambo	83.5 (80.7–86.0)	24.6 (18.7–32.2)
	Huila	35.0 (31.6–38.5)	1.5 (1.2–1.9)
	Kwanza Norte	65.0 (61.8–68.2)	6.8 (5.4–8.6)
	Kwanza Sul	25.2 (21.9–28.7)	–
	Malanje	29.9 (26.4–33.6)	1.3 (1.0–1.7)
	Uige	28.7 (26.3–31.3)	1.3 (1.1–1.6)
	Zaire	53.3 (49.8–56.7)	4.2 (3.3–5.3)
	Health facility		
	Health posts	49.7 (48.3–51.0)	–
	Health centres	50.0 (47.7–52.5)	0.8 (0.7–0.9)
	Hospitals	51.4 (44.6–58.1)	1.2 (0.9–1.7)*

All *p* values < 0.05; *p > 0.05

The lowest malaria reporting accuracy rates were registered in Kwanza Sul [25.2% (95% CI: 21.9–28.7)], whereas Huambo [83.5% (95% CI: 80.7–86.0)] showed the highest. The proportion of reports which data was matching health facility patient registers was higher in Hospitals than in health posts [51.4% (95% CI: 44.6–58.1) vs 49.7% (95% CI: 48.3–51.0)], but these differences were not found to be statistically significant. Similar proportions for the track indicator was registered between health centers and health posts. However, in multivariate analysis, health centres seemed to be less likely to report data accurately than health posts (Adj OR: 0.8; 95% CI: 0.7–0.9, *p* < 0.01).

### ACT medicines and RDT stocks

The presence of ACT medicines and RDT stocks at health facilities increased from 47.5% (95% CI: 44.8–50.2) in 2013 to 70.1% (95% CI: 68.1–72.0) in 2015 followed by a considerable fall to 64.7% (95%CI: 62.4–72.0) in 2016 (Table 6). Similar trends were verified when analyzing RDT stocks only, while the proportion with ACT medicines stocks remained relatively stable during the four years (around 80%).

**Table 6:** Proportion of health facilities with stocks of ACT and RDT, by year, province and type of health facility

Stocks	Factor	Proportion (95% CI)		
		Stocks (ACT and RDT)	Stocks of RDT	Stocks of ACT
	Year			
	2013	47.5 (44.8–50.2)	52.2 (49.5–54.9)	82.9 (80.8–84.9)
	2014	56.3 (54.0–58.6)	63.0 (60.7–65.2)	80.3 (78.4–82.1)
	2015	70.1 (68.1–72.0)	82.9 (81.3–84.5)	80.3 (78.6–81.9)
	2016	64.7 (62.4–72.0)	75.4 (73.3–77.4)	78.3 (76.3–80.2)
	Province			
	Benguela	65.1 (62.4–67.8)	75.1 (72.6–77.5)	85.7 (83.6–87.6)
	Huambo	62.6 (59.2–66.0)	75.0 (71.8–78.0)	76.3 (73.1–79.2)
	Huila	59.3 (55.7–62.8)	72.4 (69.1–75.6)	72.3 (68.9–75.4)
	Kwanza Norte	72.1 (69.1–75.0)	74.4 (71.4–77.2)	95.1 (93.5–96.4)
	Kwanza Sul	41.9 (38.1–45.8)	61.6 (57.8–65.4)	62.2 (58.4–66.0)
	Malanje	69.5 (65.8–73.0)	77.8 (74.4–80.9)	84.2 (81.2–86.9)
	Uige	51.1 (48.4–53.9)	58.9 (56.2–61.6)	72.1 (69.6–74.5)
	Zaire	66.0 (62.7–69.2)	58.9 (56.2 (61.6)	91.3 (89.2–93.1)
	Health facility			
	Health posts	60.6 (59.2–61.9)	70.0 (68.7–71.2)	80.1 (79.0–81.2)
	Health centres	60.5 (58.1–62.8)	69.0 (66.8–71.2)	80.5 (78.5–82.4)
	Hospitals	72.5 (66.1–78.2)	79.7 (73.8–84.8)	83.8 (78.2–88.4)

Kwanza Sul [41.9% (95% CI: 38.1–45.8)] and Uige [51.1% (95% CI: 48.4–53.9)] registered the lowest proportion of HF with stocks of both ACT medicines and RDT, whereas Kwanza Norte [72.1% (95% CI: 69.1–75.0)] showed the highest. ACT medicines and RDT stocks were frequently more available in hospitals than in health care posts and health centres [72.5% (95% CI: 66.1–78.2) vs 60.6% (95% CI: 59.2–61.9) and 60.5% (95% CI: 58.1–62.8), respectively].

## Discussion

Testing, treatment and tracking of malaria cases constitute the three fundamental pillars of the existing global malaria policy and strategy [2]. This study endeavoured to assess the implementation of this strategy and its implications on the management of uncomplicated malaria cases within the context of a well-supported malaria control initiative in Angola. The findings revealed major improvements in each of the three policy pillars during this period. It also revealed serious gaps, likely to undermine the National Malaria Control Strategic targets for 2016–2021.

### Knowledge

Knowledge is a critical component in the continuum of effective malaria case prevention and management. It ensures HCWs to have the right skills to apply appropriate behaviour and practices to manage malaria cases effectively [4–6]. The results demonstrate that over time, HCWs knowledge on malaria case prevention and management improved. In the context of this assessment, HCWs systematically and almost exclusively acquired knowledge through a malaria training and supervision programme that trained approximately 6500 HCWs, and undertook over 7000 supervision visits to health facilities [32].

Although this assessment did not explore any association between individual HCW’s knowledge and malaria training, there is evidence to support the association between improved knowledge and training [5–7, 13, 15, 26]. It is, therefore, plausible to infer that training and supervision had an influence on knowledge about malaria.

The results demonstrate that HCWs in Huambo were more knowledgeable than their peers in Uige. Knowledge discrepancies among HCWs between provinces are difficult to interpret, since this assessment did not explore the individual characteristics of the HCWs. However, knowledge differences among HCWs in Huambo and Zaire compared to peers in Uige and Kwanza Sul provinces, can be attributed to cumulative benefits of PMI’s malaria control interventions in Huambo and Zaire provinces, which began in 2005, compared to Uige and Kwanza Sul which were only initiated in 2011 [33]. Interestingly, a study that assessed the associated between HCWs training and malaria case management practices, between Uige and Huambo observed stark differences in the state of malaria case management [25], with testing rates of suspect malaria cases in Huambo at 30% (range: 23–38) vs 69% (53–81) in Uíge,and overall, 28% (13–49) of patients with uncomplicated malaria, appropriately treated with an ACT medicines with the correct dose in Huambo, compared to 60% (42–75) in Uíge. It also observed an association between incorrect case management of suspect malaria cases with lack of healthcare worker training in Huambo, compared to ACT medicines stock-outs in Uíge. The observations highlight the need for further studies designed to establish the underlying factors that determine inter-provincial variations related to malaria case management, given the implications they have on malaria programme implementation.

### Testing and treating

The results observed 2 critical findings. The 2 important components in the continuum of malaria case management and the 3 T policy—testing suspected malaria case with RDTs and treating confirmed cases with approved ACT medicines—improved significantly overtime among HCWs.

In the absence of any other malaria case management quality improvement interventions, within the context of this programme, one can infer that cumulative benefits of training and supervision can affect these results. Also, it should not be excluded that experience gained by HCW over time may have played a significant role in the improvements noted. Importantly the results demonstrate a steady increase in the proportion of health facilities that test and treat over 75% of malaria cases over time as training and supervision visits were implemented. These findings are consistent with previous research that found an association between training and supervision and improved testing and treatment practices [15, 24–26, 34].

Although hospitals tested and treated a significantly higher proportion of malaria cases, compared to other health facilities, improvements in health posts are noteworthy, because health posts constitute approximately 70% of all health facilities, and tend to serve rural populations, which carry the highest burden of malaria [35]. Any improvements at this level are thus likely to have a significant impact on malaria control efforts.

Additionally, the results demonstrate that hospitals suffered less stock-outs of RDTs and ACT medicines compared to other health facilities and, therefore, HCWs in hospitals are likely to test and treat more malaria cases [3, 36]. Despite the low coverage of microscopy in some of the provinces assessed [25], the existence of an alternative to RDT may explain better testing rates in hospitals who tend to be better equipped and staffed. There was however no evidence to show why hospitals experienced less stock-outs of RDTS and ACT medicines compared to other health facilities, since the availability of RDTs and ACT increased significantly throughout Angola during the programme period [37]. Better qualified HCWs in hospitals compared to health posts likely has a bearing on these findings [35], but the remoteness and condition of accesses to some of these local HF may certainly have been responsible for some of the stock-outs verified.

Targeted investments to strengthen malaria commodity supply chain systems at national, provincial, municipal and health facility levels were undertaken during this period that should likely have had a bearing on the effectiveness of malaria commodity supply chain systems [38]. These findings underscore the need to identify the underlying causes of stock outs of essential anti-malaria commodities and find locally appropriate solutions that can help overcoming this issue particularly within different categories of health facilities. Supporting effective communication between neighbouring HF may help improve stock management and have broader impact in malaria case management [39].

The variations in testing and treatment rates between HCWs in different provinces are difficult to establish, especially when assessed against similar malaria transmission epidemiological profiles, trainings and supervisions. Stark differences between the general quality of health care services between Uige and Luanda provinces may be attributed to historical, social, cultural and economic factors [13]. The general mind set of HCWs in relation to testing and treating malaria cases with RDTs and ACT medicines was also identified as a potential factor determining HCW behaviour and practices in Uige and Huambo provinces [25]. In Benguela, anecdotal evidence on HCW distrust of RDTs results was also identified as a major problem for accurate malaria diagnosis [40], confirming previous evidence generated in Luanda about malaria over diagnosis and over prescription with [20].

These findings highlight the importance of sustaining efforts in training and supervision for correct malaria diagnosis and treatment. It is essential to ensure HCW have access to adequate training that focus on major RDT misperceptions and understand the value that RDT results bring to inform the diagnosis. Overcoming most common wrong perceptions and attitudes on malaria case management is essential [17]. However, broader systemic issues like continuous supervision and stock supply should not be forgotten. This is particularly important at lower levels of health care provision where less trained health staff tend to wrongly prescribe anti-malarial drugs without testing for malaria [12].

### Malaria case registration and tracking

Malaria data is critical to accurately measure the effectiveness of interventions against malaria and increasingly assess value for money, especially now that resources for malaria are progressively decreasing [41]. As primary sources of malaria tracking data, health facility registers need to be accurate. The measure of accuracy used focused on the correctness between health facility monthly malaria reports and health facility patient registers for a similar period. This assessment noted that malaria data accuracy did not demonstrate significant improvements compared to those observed for testing and treatment—despite receiving similar quality improvement interventions. Previous reports in Angola also identified malaria data accuracy as a problem [40].

With only 55% of malaria reports correct in 2016, these results indicate that training and supervision of HCWs alone does not necessarily improve the correctness of malaria data across all categories of health facilities. Since these 2 interventions constitute the main components of quality improvement interventions for malaria control programs, more comprehensive assessments to explore factors that affect and determine malaria data quality are critical, to enable the design of more evidence-based interventions.

## Limitations of the study

The study did not draw comparisons with a control group of provinces in Angola with similar malaria epidemiological profiles that never benefited from the malaria control initiative. Although that was not the scope of this assessment, a comparison would have strengthened the power and generalizability of the findings, and informed on-going policy discussions aimed at extending and broadening the scope of this programme in selected provinces over the next five years. Since data was not collected in Huambo in 2016, temporal comparisons are naturally affected by this limitation.

Secondly, data collection was designed to answer programmatic needs. These proved to be a limitation in commuting some variables into values that would be more appropriate to conduct further data analysis. The non-inclusion of information on number of supervision and trained health staff into data analysis is a major limitation that undermined the possibility to measure the real impact of these activities in the results found.

Potential bias in data collection introduced by different supervisors in different provinces may have existed. To overcome this problem, a rigorous system of supervisor’s evaluation was put in place to ensure all complied with data collection guidelines. These monitoring visits were conducted annually to ensure regular follow up and evaluation of procedures. A potential Hawthorne effect may have happened as supervision visits were planned in advance and health workers had time to prepare for the supervision. Results reading should consider this potential source of bias. However, findings still point out several gaps in the system. Therefore, this bias may be only underestimating the potential gaps still existing in the system.

## Conclusion

Significant improvements in the quality of uncomplicated malaria case management were observed, particularly related to testing and treatment of malaria cases at all levels of health care delivery. Tracking of malaria cases continues to pose a challenge, despite on-going efforts to improve malaria data quality. The improvements registered in knowledge about malaria in this assessment seemed to have translated into better testing and treatment practices among HCWs.

Hospitals continue to perform better in all parameters compared to other health facilities, and more efforts are required to bridge this gap. To sustain the gains attained within this initiative, and to improve coverage, intervention programmes must adopt a health system approach towards resolving barriers that hamper optimal coverage. Research and regular assessments must accompany programme implementation to gather more evidence and provide adequate guidance.

## Supplementary information

**Additional file 1.** Indicators collected during formative supervision visits.

## Abbreviations

ACT: Artemisinin-based combination therapy
AL: Artemether/lumefantrine
RDT: Rapid diagnostic tests
NGO: Non Governmental Organization
PMI: United Sates President’s Malaria Initiative
WHO: World Health Organization
