# Supplementary material for: Malaria Test, Treat and Track policy implementation in Angola: a retrospective study to assess the progress achieved after 4 years of programme implementation
Source: Malar J. 2020 Jul 20;19:262. doi: 10.1186/s12936-020-03338-x (PMC7372868; doi:10.1186/s12936-020-03338-x)
Supplement: Supplementary file 1 — Additional file 1. Indicators collected during formative supervision visits. [file 12936_2020_3338_MOESM1_ESM.docx]

**Aditional File 1** – Indicators collected during Formative Supervision visits

| **Questions/Indicators** | **Assessment Criteria used** | **Variable Values** |
| --- | --- | --- |
| 1 - Health worker knows how to perform a RDT | The Health worker complies with all of the following:  1 – Follows biosafety procedures;  2 – Applies blood and buffer in appropriate RDT place  3 – Does a correct interpretation of results | 1 – The health worker knows how to do a RDT  Or  2 - The health worker doesn’t know how to do a RDT |
| 2 - Health worker understands what is differential diagnosis of fever | The Health worker identifies both:  1 – The importance of looking other causes of fever when malaria RDT is negative  2 – Other potential causes of fever and associated signs and symptoms to look for | 1 – The health worker knows what is differential diagnosis of fever  Or  2 - The health worker does not know what is differential diagnosis of fever |
| 3 – The Health Worker knows correct dosage and posology of ACT | The Health worker identifies both:  1 – The correct dose for different weights (identifies at least 2 different weight intervals and respective ACT dosage)  2 – The correct timing between ACT uptakes identifying the importance of completing 3 days of treatment | 1 – The health worker knows correct dosage and posology of ACT  Or  2 - The health worker does not know correct dosage and posology of ACT |
| 4 – The Health Worker knows when Intermittent Preventive Treatment in pregnancy (IPTp) should start | The Health worker identifies either:  1 – When the women first feels foetal movements  Or  2 – From second trimester of pregnancy onwards  Or  3 – From 16^th^ week of pregnancy onwards. | 1 - The Health Worker knows when Intermittent Preventive Treatment in pregnancy (IPTp) should start  Or  2 – The Health Worker does not know when Intermittent Preventive Treatment in pregnancy (IPTp) should start |
| 5 – Case confirmation of malaria suspected cases either with microscopy or RDT | By consulting the previous 30 day registers in Health Facility, calculate percentage using:  Numerator: Number of suspected malaria cases (registered as such or registered as febrile cases) tested with RDT or microscopy  Denominator: Number of suspected malaria cases (registered as such or registered as febrile cases) | 1 – Less than 75% of suspected cases tested  2 –75% or more of suspected cases tested |
| 6 – Non severe malaria cases treated with first line malaria medicine (ACT) | By consulting the previous 30 day registers in Health Facility, calculate percentage using:  Numerator: Non severe malaria cases treated with ACT  Denominator: All non-severe malaria cases registered. | 1 – Less than 75% of non-severe cases treated with ACT  2 - 75% or more of non-severe cases treated with ACT |
| 7 – Health Unit Facing RDT or ACT stock outs | By consulting Health Facility Stock Registers consider:  1 - In the last 3 months a period superior to 7 days with ACT stock outs,  And/or  2 - In the last 3 months a period superior to 7 days with RDT stock outs | 1 – No stock outs  2 – Stock out of RDT  3 – Stock out of ACT  4 – Stock Out of RDT and ACT |
| 8 – Health Facility Malaria monthly report data agrees with Health Facility register book | By consulting registration books in Health Facility, compare the number of reported cases and deaths in monthly report against the data in registration books and check if there is any discrepancy or data gap. | 1 – Monthly Malaria report data is in agreement with Health Facility registration book  2 - Monthly Malaria report data is not in agreement with Health Facility registration book or is not available for analysis |
